# Development and validation of a short version of the Assessment of Chronic Illness Care (ACIC) in Dutch Disease Management Programs

**DOI:** 10.1186/1477-7525-9-49

**Published:** 2011-07-04

**Authors:** Jane M Cramm, Mathilde MH Strating, Apostolos Tsiachristas, Anna P Nieboer

**Affiliations:** 1 Institute of Health Policy & Management (iBMG). Erasmus University Rotterdam, The Netherlands

**Keywords:** chronic care, measurement, quality, chronic illness, disease management

## Abstract

**Background:**

In the Netherlands the extent to which chronically ill patients receive care congruent with the Chronic Care Model is unknown. The main objectives of this study were to (1) validate the Assessment of Chronic Illness Care (ACIC) in the Netherlands in various Disease Management Programmes (DMPs) and (2) shorten the 34-item ACIC while maintaining adequate validity, reliability, and sensitivity to change.

**Methods:**

The Dutch version of the ACIC was tested in 22 DMPs with 218 professionals. We tested the instrument by means of structural equation modelling, and examined its validity, reliability and sensitivity to change.

**Results:**

After eliminating 13 items, the confirmatory factor analyses revealed good indices of fit with the resulting 21-item ACIC (ACIC-S). Internal consistency as represented by Cronbach's alpha ranged from 'acceptable' for the 'clinical information systems' subscale to 'excellent' for the 'organization of the healthcare delivery system' subscale. Correlations between the ACIC and ACIC-S subscales were also good, ranging from .87 to 1.00, indicating acceptable coverage of the core areas of the CCM. The seven subscales were significantly and positively correlated, indicating that the subscales were conceptually related but also distinct. Paired t-tests results show that the ACIC scores of the original instrument all improved significantly over time in regions that were in the process of implementing DMPs (all components at *p *< 0.0001).

**Conclusion:**

We conclude that the psychometric properties of the ACIC and the ACIC-S are good and the ACIC-S is a promising alternate instrument to assess chronic illness care.

## Introduction

The increasing prevalence of the chronically ill due to population aging and longevity [1] has resulted in deficiencies in the organization and delivery of care [2-4]. Accumulated evidence shows under-diagnosis, under-treatment, and failure to use primary and secondary prevention measures [5,6] among the chronically ill. There is also evidence that interventions and quality improvements in organizational and clinical processes of primary care can improve such care [7-12]. The literature strongly suggests that changing processes and outcomes in chronic illness requires multicomponent interventions [12-14].

Disease management programs (DMPs) aim to improve effectiveness and efficiency of chronic care delivery [15]. In the literature there are basically two types of disease management models: (1) commercial DMPs and (2) primary care DMPs aiming to improve quality of chronic care based on the Chronic Care Model (CCM) [16]. Commercial DMPs are the oldest models and are more common in the United States. The commercial service is contracted by a health plan to provide selected chronic disease assessment and educational services by telephone, usually for a single condition. Commercial DMPs provide care to chronically ill patients without any involvement of regular primary and hospital care [17]. These commercial DMPs are contracted and paid by health insurance companies. The other type of DMPs are based on the chronic care model (CCM) introduced by Edward Wagner [1]. The CCM was developed as a foundation for the redesigning of primary care practices and forms the basis for effective chronic-care management. It addresses shortcomings in acute care models by identifying essential elements that encourage high-quality chronic-disease care [11,12].

DMPs in the Netherlands are based on the CCM. This model provides an organised multidisciplinary approach to the delivery of care for patients with chronic diseases, which involves the community and the healthcare system and fosters communication between clinicians and well-informed patients. Unlike the commercialized DMPs targeting patients only, DMPs based on the CCM are aimed at patients as well as professionals [18].

The CCM clusters six interrelated components of health care systems: health care organization, community linkages, self-management support, delivery system design, decision support and clinical information systems. The idea is to transform chronic disease care from acute and reactive to proactive, planned, and population-based [1]. Of the six components, the self-management component relies heavily on community-based resources, including rehabilitation programmes, patient-education materials, group classes, and ideally a home health-case manager who can regularly assess difficulties and acknowledge accomplishments. The delivery-system design component of the CCM requires well-trained clinical teams that ensure successful self-management, coordinate preventive care, screen for common comorbidities, and address questions or acute issues around the clock. An active clinical information system provides clinicians with performance feedback and automated reminders of practice guidelines. Finally, the decision support component involves the use of evidence-based practice guidelines, which are critical for the optimal management of any chronic illness. Effective management of complex chronic diseases is best accomplished by collaboration among clinicians with the support of a variety of healthcare resources.

The Assessment of Chronic Illness Care (ACIC, see appendix 1) is based on six areas of system change suggested by the CCM and was developed to help disease-management teams identify areas for improvement in chronic illness care and evaluate the level and nature of improvements made in their system [11,14,19-21]. The ACIC is one of the first comprehensive tools targeting generic organization of chronic care across disease populations, rather than traditional disease-specific tools such as HbA1c levels, productivity measures (e.g., number of patients seen), or process indicators (e.g., percentage of diabetic patients receiving foot exams). The ACIC attempts to represent poor to optimal organization and support of care in the CCM areas [21].

Research shows that the ACIC appears sensitive to interventions across chronic illnesses and helps teams focus their efforts on adopting evidence-based chronic care changes. As such the ACIC represents a useful tool to investigate the progress of DMPs over time. Overall however, the literature base for the ACIC is extremely limited, with no previously published studies providing an in-dept investigation of the ACIC's psychometric qualities. Therefore, we investigated the psychometric properties of the ACIC. The cumbersome length of the ACIC led us to additionally perform an item reduction analysis and develop a short version. A short version of the ACIC makes it less burdensome for professionals to fill in the questionnaire and therefore easier to assess chronic care delivery.

In this article, we describe the psychometric testing of the ACIC in 22 DMPs participating in quality improvement projects focused on chronic care in the Netherlands. Our objectives are to validate the original 34-ACIC and to reduce the number of items of the original 34-item ACIC while maintaining validity, reliability, and sensitivity to change.

## Methods

Our study was performed with professionals of DMPs teams in the Netherlands. These DMPs consist of a variety of collaborations (mostly general practitioners, physiotherapists, dieticians) undergoing internal practice redesign to improve effective chronic-care management. The DMPs address shortcomings in acute care models by identifying essential elements that encourage high-quality chronic-disease care. These DMPs are initiated and controlled by the practices. Due to the importance of changing acute primary care into high-quality chronic-disease care a national programme on "disease management of chronic diseases" carried out by ZonMw (Netherlands Organisation for Health Research and Development) and commissioned by the Dutch Ministry of Health, provided funding for practices planning a redesigning of primary care according to the CCM. Requirements of the national programme were that the practices had to have some experience with the delivery of chronic care and were equipped to implement all systems needed for the delivery of sufficient chronic care, which resulted in the inclusion of 22 DMPs (out of 38). These DMPs can be considered to be among the leaders of chronic care delivery in the Netherlands. We evaluated 22 DMPs that aimed to enhance knowledge on disease-management experience in chronic disease care and stimulate implementation of successful programs [22]. The primary aim of our evaluation is to get information about the quality of the DMPs and their alignment with the CCM as well as on the improvement over time after implementation.

The DMPs were implemented in various Dutch regions. The DMPs targeted several patient populations: cardiovascular diseases (9), chronic obstructive pulmonary disease (COPD) (5), diabetes (3), heart failure (1), stroke (1), depression (1), psychotic diseases (1), and eating disorders (1). The intervention concerned the implementation of DMPs. Each DMP consisted of a combination of patient-related, professionally-directed and organizational interventions. The exact programme components for each region may vary. The core of a DMP is described below; for detailed programme information, see our study protocol [22].

### Patient-related interventions

Self-care is critical to optimal management of chronic diseases. Hence, all 22 DMPs included such interventions. Examples of self-management within the DMPs are patient education on lifestyle, regulatory skills, and proactive coping.

### Professional-directed interventions

Care standards, guidelines, and protocols are essential parts of the 22 DMPs. They are integrated through timely reminders, feedback, and other methods that increase their visibility at the time that clinical decisions are made. All DMPs are built on these (multidisciplinary) guidelines. The implementation strategies for professional interventions may, however, vary. All DMPs provide training for their professionals. Implementation of these guideline in 19 DMPs was supported by ICT tools such as integrated information systems.

### Organisational interventions

Many forms of organisational changes are applied in the 22 DMPs. Examples of organisational interventions are new collaborations of care providers, allocating tasks differently, transferring information and scheduling appointments more effectively, case management, using new types of health professionals, redefining professionals' roles and redistributing their tasks, planned interaction between professionals, and regular follow-up meetings by the care team.

### Participants

In 2009 the national programme on "disease management of chronic diseases" selected 22 DMPs for funding. During this initial phase of the program we learned that the DMPs faced many barriers to implement their DMPs. Changing the approach toward patient-centeredness and more support for self-management demands a lot on the part of the organization and professionals, as well. Organizing and training health care providers to implement the DMP is time-consuming on the part of the project leaders and the health care providers. Training the GPs, overseeing the implementation of the DMP at the provider level, and assisting with challenges for health care offices can take more time than was planned in the project plans. Therefore, we only approached the core DMP team to establish the level of chronic care delivery in 2009. The core team of the DMPs mainly consisted of project leaders and physicians (total of 142). Response rate of the baseline measurement was 63 percent: eighty-nine respondents filled in the questionnaire at T0 (consisting of the four main components of the CCM only). A year later (2010) most DMPs finished implementing the interventions of their DMP (e.g. ICT-systems, training professionals) and started including patients. A questionnaire (T1) was sent to all 393 professionals participating within the 22 DMPs. A total of 218 respondents filled in the questionnaire (response rate 55 percent). Fifty-three respondents filled in the questionnaires at both T0 and T1.

Either a package of questionnaires was sent to the contact person of each participating organization (which were distributed to potential respondents through their mail boxes or delivered personally at team meetings) or questionnaires were sent directly to the potential respondents. Two weeks later the same procedure was used to send a reminder to non-respondents. No incentives in the form of money or gifts were offered.

### Measures

The current ACIC consists of 34 items covering the six areas of the CCM: health care organization (6 items); community linkages (3); self-management support (4); delivery system design (6); decision support (4); clinical information systems (5). The ACIC also covers integrating the six components, such as linking patients' self-management goals to information systems (6 items) [23]. After obtaining permission to use and translate the ACIC from the The MacColl Institute for Healthcare Innovation, Group Health Cooperative we followed a translation approach. An official native translator and two research team members independently translated the English ACIC version into Dutch. The research group reconciliation was carried out into a single forward translation. The back translator translated the ACIC Dutch version back into the source language. The project team compared both versions and discussed the professionals' comments and issues that caused confusion. This process led to the final version of the Dutch-ACIC, the D-ACIC.

Responses to ACIC items (e.g., "Evidence-based guidelines are available and supported by provider education") fall within four descriptive levels of implementation ranging from ''little or none'' to a ''fully-implemented intervention''. Within each of the four levels, respondents are asked to choose the degree to which that description applies. The result is a 0-11 scale, with categories defined as: 0-2 (little or no support for chronic illness care); 3-5 (basic or intermediate support for chronic illness care); 6-8 (advanced support); and 9-11 (optimal, or comprehensive, integrated care for chronic illness). Subscale scores for the six areas are derived by summing the response choices for items in that subsection and dividing it by the corresponding number of items. Bonomi and colleagues [20] have shown the six ACIC subscale scores to be responsive to health care quality-improvement efforts.

Reliability of the instrument was assessed by determining the statistical coherence of the scaled items, which reflects the degree to which they measure the intended aspect of chronic care. Validity is the degree to which a scale measures what it is intended to measure; here we focused on the construct validity of the questionnaire and sensitivity to change.

### Analysis

Our analyses involved the following seven steps.

1. The sample characteristics were analysed using descriptive statistics.

2. We data-screened the items by examining the number of missing and not applicable responses, and the mean and standard deviation of each item.

3. To verify the factor structure of the questionnaire and test for the existence of the relationship between observed variables and their underlying latent constructs, we executed confirmatory factor analysis using the LISREL program [24]. No correlation errors within or across sets of items were allowed in the model.

4. Item reduction analysis was performed to develop a short version of the questionnaire. Items removal followed three criteria: (i) items were excluded following modification indices provided by LISREL and the strength of the factor loadings; (ii) item elimination was stopped when reliability of each subscale dropped below 0.70; and (iii) as many items as possible were eliminated (minimum = 3) without loss of content and psychometric quality. Listwise deletion of cases with missing data on the 34 items resulted in N = 110. To test the measurement models, we used four indices of model fit whose cut-off criteria were proposed by Hu and Bentler [25]. First, the overall test of goodness-of-fit assessed the discrepancy between the model implied and the sample covariance matrix by means of a normal-theory weighted least-squares test. A plausible model has low, preferably non-significant χ^2 ^values. However, Chi-square is overly sensitive in a large sample (over 200) [26], leading to difficulty in obtaining the desired non-significant level [27]. Second, the Root Means Square Error of Approximation (RMSEA) reflects the estimation error divided by the degrees of freedom as a penalty function. RMSEA values below 0.06 indicate small differences between the estimated and observed model. Third, we used the Standardized Root Means square Residual (SRMR), which is a scale-invariant index for global fit ranging between 0 and 1. SRMR values below 0.08 indicate a good fit. Fourth, we calculated the Incremental Fit Index (IFI), which compares the independent model (i.e., observed variables are unrelated) to the estimated model. IFI values are preferably larger than 0.95.

5. The final Dutch ACIC-S was tested on an imputed dataset by replacing missing values with the mean of each DMP team as scored by the other professionals of the same DMP team, resulting in N = 218, or the total sample.

6. Internal consistency of the subscales was assessed by calculating Cronbach's alphas, inter-item correlations within each subscale, and correlations between subscales.

7. We investigated the sensitivity to change of the original ACIC and the ACIC-S to assess its ability to accurately detect changes. Data sources used were (i) pre-post, self-report ACIC data from the initiators of the 22 projects enrolled in the national programme on "disease management of chronic diseases" and (ii) self-report ACIC data from all professionals of all DMP teams one year after the DMPs' implementation. Since at the time the DMPs were not yet fully implemented and DMP teams not yet fully formed, only the initiators of each DMP were asked to rate the level of chronic illness care congruent with the four main components of the CCM, i.e., 'self-management support', 'delivery system design', 'decision support', and 'clinical information systems'. Paired t-tests were used to evaluate the sensitivity of the ACIC and ACIC-S to detect system improvements for DMP teams in the 22 DMPs focused on cardiovascular diseases, COPD, diabetes, heart failure, stroke, depression, psychotic diseases, and eating disorders.

## Results

### Sample characteristics

Table 1 displays descriptive characteristics of the sample of professionals. Of those completing the questionnaire in 2010 (response rate 55 percent, 218/393), the majority was female (66 percent) and mean age was 47.2 years (sd 9.47), ranging from 25 to 65. About 75 percent had been working for more than three years within the organisation. More than half (67 percent, 144) worked more than 29 hours per week. DMP teams mainly consisted of general practitioners (35 percent), practice nurses (29 percent), policy/management (13 percent) and para/perimedical professionals (12 percent).

**Table 1:** Sample characteristics professionals (n = 218)

		**No**.	Percentage
Gender	- female	139	66.2%
	- male	71	33.8%
Working past	- more than 3 years	160	75.1%
Working hours	- more than 29 hours	144	67.6%
Occupation	- General Practitioner	76	34.9%
	- practice nurses	56	25.7%
	- policy and management	28	12.8%
	- para-/perimedical professionals	26	11.9%
	- medical/social specialists	6	2.8%
	- others	26	11.9%

No. = Number of respondents

### Datascreening

All items were screened for univariate and bivariate normality, and to detect outliers. No extreme values were found. Some items had a relatively high number of missing data and 'not applicable' answers, in particular those under ICT and integration (table 2). Data screening information was taken into account in the stepwise procedure of the item reduction analysis.

**Table 2:** Item characteristics and factor loadings of the first full model

Item		missing	not applicable	mean	sd	λ
**Organization of the Healthcare Delivery System**						
1. Overall organizational leadership in chronic illness care	211	7 (3.2%)	4 (1.8%)	7.38	2.36	.80
2. Organizational goals for chronic care	212	6 (2.8%)	4 (1.8%)	7.58	2.18	.88
3. Improvement strategy for chronic illness care	210	8 (3.7%)	7 (3.2%)	6.98	2.35	.81
4. Incentives and regulations for chronic illness care	207	11 (5.0%)	10 (4.6%)	6.84	2.49	.73
5. Senior leaders	209	9 (4.1%)	15 (6.9%)	8.24	2.16	.62
6. Benefits	204	14 (6.4%)	13 (6.0%)	6.66	2.73	.66

**Community linkages**						
7. Linking patients to outside resources	208	10 (4.6%)	7 (3.2%)	6.23	2.53	.62
8. Partnership with community organizations	209	9 (4.1%)	5 (2.3%)	7.16	2.11	.75
9. Regional health plans	206	12 (5.5%)	26 (11.9%)	7.22	2.57	.88

**Self-management support**						
10. Assessment and documentation of self-management needs and activities	209	9 (4.1%)	1 (0.5%)	5.85	2.78	.82
11. Self-management support	210	8 (3.7%)	4 (1.8%)	6.44	2.97	.87
12. Addressing concerns of patients and families	210	8 (3.7%)	2 (0.9%)	6.49	2.07	.78
13. Effective behavior change interventions and peer support	208	10 (4.6%)	4 (1.8%)	7.07	2.46	.73

**Decision support**						
14. Evidence-based guidelines	210	8 (3.7%)	3 (1.4%)	7.88	1.79	.74
15. Involvement of specialists in improving primary care	209	9 (4.1%)	4 (1.8%)	6.79	2.80	.68
16. Providing education for chronic illness care	208	10 (4.6%)	6 (2.8%)	6.66	2.42	.78
17. Informing patients about guidelines	209	9 (4.1%)	3 (1.4%)	6.22	2.50	.76

**Delivery system design**						
18. Practice team functioning	206	12 (5.5%)	5 (2.3%)	6.72	2.19	.78
19. Practice team leadership	206	12 (5.5%)	4 (1.8%)	7.09	2.33	.67
20. Appointment system	206	12 (5.5%)	6 (2.8%)	6.31	2.22	.69
21. Follow-up	209	9 (4.1%)	2 (0.9%)	7.39	2.30	.73
22. Planned visits for chronic illness care	209	9 (4.1%)	3 (1.4%)	8.78	1.84	.67
23. Continuity of care	207	11 (5.0%)	2 (0.9%)	7.45	2.11	.79

**Clinical information systems**						
24. Registry (list of patients with specific conditions)	207	11 (5.0%)	9 (4.1%)	6.74	2.31	.63
25. Reminders to providers	203	15 (6.9%)	21 (9.6%)	5.92	3.60	.46
26. Feedback	207	11 (5.0%)	12 (5.5%)	6.51	2.53	.65
27. Information about relevant subgroups of patients needing services	202	16 (7.3%)	9 (4.1%)	6.37	2.54	.71
28. Patient treatment plans	208	10 (4.6%)	3 (1.4%)	6.35	2.68	.79

**Integration of chronic care components**						
29. Informing patients about guidelines	207	11 (5.0%)	6 (2.8%)	6.24	2.46	.78
30. Information systems/registries	204	14 (6.4%)	12 (5.5%)	5.13	3.15	.73
31. Community programs	205	13 (6.0%)	34 (15.6%)	5.79	3.62	.71
32. Organizational planning for chronic illness care	204	14 (6.4%)	10 (4.6%)	5.69	2.50	.76
33. Routine follow-up for appointments patient assessments and goal planning	206	12 (5.5%)	10 (4.6%)	6.96	2.40	.74
34. Guidelines for chronic illness care	206	12 (5.5%)	8 (3.7%)	5.40	2.78	.89

### Confirmatory Factor analysis with 34 items

All items had factor loadings above 0.60 except for item 25, which was 0.46. Standardized loadings of the items are shown in table 2. Indices of model fit showed sufficiency (table 3 model 1). The significant Normal Theory Weighted Least Square χ^2 ^statistic of 1022.22 is not surprising given its sensitivity to sample size. The RMSEA was just above cut-off value but, according to criteria of Hu and Bentler [24], acceptable. IFI was above cut-off value of 0.95 and SRMR was below the cut-off value of 0.08. All indices indicated that the model was acceptable, but left room for improvement and shortening.

**Table 3:** Model fit of the full and short models

	Χ^2 ^(p)	RMSEA	IFI	SRMR
Model 1: 34 items (n = 110)	1022.22 (0.00)	0.0687	0.979	0.0696
Model 2: final short version (n = 110)	286.70 (0.00)	0.0510	0.991	0.0620
Model 3: final short version on imputed data (n = 218)	306.115	0.0616	0.980	0.0501

### Item reduction analysis

Following the factor loadings, modification indices, and the internal consistency check of each subscale, the stepwise procedure resulted in elimination of 13 items^1^. The final short version consisted of 21 items, or three items per subscale. The overall fit of this final model was improved as compared with the 34-item version (table 3, model 3). The Normal Theory Weighted Least Square χ^2 ^significantly decreased to 286.70; RMSEA at 0.05 was below the cut-off point of 0.06; and the IFI value of 0.99 indicated that the specified relations between variables were well supported by the data. The SRMR index decreased to 0.0620 (still below the cut-off point of 0.08), indicating a good global fit of the overall model. The final short model on imputed data resulted in comparable factor loadings and its model indices showed good fit.

### Internal consistency and inter-correlations

Internal consistency as represented by Cronbach's alpha ranged from acceptable ('clinical information systems' subscale) to excellent ('organization of the healthcare delivery system' subscale) (table 4). The correlations between the full original subscales and short subscales were good, ranging from 0.87 to 1.00, indicating acceptable coverage of the core areas of the CCM (table). The seven subscales were significantly and positively correlated (table 4), indicating conceptually-related subscales.

**Table 4:** Scale characteristics and inter-correlations of the shortened subscales (n = 218)

	items short version	Cron-bach's alpha	original full scale	scale mean (sd)	inter-item correlations range	1	2	3	4	5	6
1. Organization of the healthcare delivery system	1,2,3	0.86	0.93**	21.71 (5.72)	.60-.70	-					
2. Community linkages	7,8,9	0.74	1.00**	19.66 (4.99)	.46-.56	0.55**	-				
3. Self-management support	10,11,12	0.79	0.97**	18.61 (6.47)	.51-.65	0.50**	0.49**	-			
4. Decision support	14,16,17	0.73	0.95**	20.57 (5.20)	.48-.50	0.50**	0.55**	0.61**	-		
5. Delivery system design	21,22,23	0.72	0.88**	23.47 (4.96)	.42-.54	0.53**	0.52**	0.61**	0.62**	-	
6. Clinical information systems	26,27,28	0.70	0.87**	18.35 (5.64)	.32-.55	0.50**	0.44**	0.67**	0.56**	0.64**	-
7. Integration of chronic care components	29,33,34	0.79	0.91**	17.84 (5.83)	.48-.68	0.51**	0.43**	0.67**	0.70**	0.62**	0.68**

** p < 0.01 (1-tailed)

### Sensitivity to change

We investigated the sensitivity to change of the four core components (self-management support, delivery system design, decision support, clinical information systems) in the original ACIC and the ACIC-S to assess its ability to accurately detect changes if they occurred. Unfortunately, one item of the decision support subscale ('informing patients about guidelines') was missing in the baseline measurement. Eighty-nine professionals filled in the questionnaire at T0 and fifty-three respondents filled in the questionnaires at both T0 and T1.

The average baseline scores across all DMPs at the beginning of the project ranged from 4.91 (clinical information systems) to 6.18 (delivery system design) indicating basic to reasonably good support for chronic illness care. Table 5 shows that the Dutch DMPs had better results in most subscales than the baseline scores measured by Bonomi and colleagues [20] and Swiss scores [28]. Requirements of the national programme of "disease management of chronic diseases" were that the practices had to have some experience with the delivery of chronic care and were equipped to implement all systems needed for the delivery of sufficient chronic care. This could explain the slightly higher scores on delivery system design, decision support, and clinical information systems as compared with Bonomi and colleagues and the Swiss scores.

**Table 5:** Average ACIC scores comparison between the 22 DMPs in the Netherlands (n = 218), Swiss primary care organisations (n = 25) and average ACIC scores at start of Chronic Care Collaboration tested by Bonomi et al., 2002 (n = 90)

ACIC Subscale Scores								
	**Self-management**		**Decision support**		**Delivery system design**		**Information systems**	

**Samples**	**M**	**SD**	**M**	**SD**	**M**	**SD**	**M**	**SD**
Swiss primary care organisations	4.71	(1.29)	4.07	(1.17)	4.96	(1.72)	3.20	(1.80)
Overall baseline scores Bonomi	5.41	(2.00)	4.80	(1.99)	5.40	(2.23)	4.36	(2.19)
Dutch disease management programmes	5.15	(1.99)	5.61	(1.94)	6.18	(1.70)	4.91	(1.80)

All four ACIC subscale scores were responsive to system improvements. Paired t-tests results showed that the ACIC scores of the original instrument all improved significantly at *p *< 0.001 (table 6). We also tested the sensitivity to change of the ACIC-S. Paired t-tests results also showed that the scores improved significantly (all at *p *< 0.001) (Table 7). The most substantial improvements measured by the original ACIC and ACIC-S were in self-management. After implementation, scores across all DMPs ranged from 6.25 and 6.78 (clinical information systems) to 7.52 and 7.97 (delivery system design) as measured by the original ACIC and the ACIC-S respectively, indicating reasonably good support for chronic care regardless the instrument used.

**Table 6:** Sensitivity to change of the original ACIC (n = 53)

	Baseline assessment		Follow-up assessment		Original ACIC change scores (T1-T0)		Significance of difference^a^
	
	M	SD	M	SD	M	SD	*P*-value
Self-management support	5.15	(1.99)	7.03	(1.82)	1.89	(2.07)	< 0.0001
Decision support	5.61	(1.94)	7.13	(1.86)	1.52	(2.44)	< 0.0001
Delivery system design	6.18	(1.70)	7.52	(1.31)	1.34	(2.08)	< 0.0001
Clinical information systems	4.91	(1.80)	6.25	(1.53)	1.34	(2.29)	< 0.0001

^a ^Significance of difference between original ACIC scores at baseline and follow-up. Paired t-tests were used to test significance of difference.

**Table 7:** Sensitivity to change of the ACIC-S (n = 53)

	Baseline assessment		Follow-up assessment		Original ACIC change scores (T1-T0)		Significance of difference^a^
	
	M	SD	M	SD	M	SD	*P*-value
Self-management support	4.85	(2.09)	6.88	(1.89)	2.06	(2.20)	< 0.0001
Decision support	6.03	(1.94)	7.40	(1.51)	1.37	(2.05)	< 0.0001
Delivery system design	6.33	(1.82)	7.97	(1.36)	1.64	(2.19)	< 0.0001
Clinical information systems	5.07	(2.13)	6.78	(1.76)	1.71	(2.60)	< 0.0001

^a ^Significance of difference between ACIC-S scores at baseline and follow-up. Paired t-tests were used to test significance of difference.

## Discussion

This study aimed to validate the original ACIC in the Netherlands as an instrument to evaluate the level and nature of improvements made by DMPs. The ACIC is a comprehensive tool specifically focused on organization of care for chronic illnesses as opposed to traditional outcome measures [11,14,20,21]. This is the first study to evaluate the level and nature of improvements made in 22 DMPs participating in quality improvement initiatives focused on chronic illness care in the Netherlands. The confirmatory factor analysis, internal consistency, inter-correlations and sensitivity to change analyses with 34 items showed that the psychometric properties of the original ACIC are satisfactory. Baseline scores were generally similar across teams addressing different chronic illnesses, and consistently showed improvement after interventions across CCM elements.

The cumbersome length of the ACIC, however, led us to perform an item reduction analysis and develop a short version (ACIC-S). The results of the confirmatory factor analyses revealed good indices of fit with the ACIC-S. As indicated by the high reliability coefficient, the scale showed good internal consistency. In case the original ACIC is considered too lengthy, the ACIC-S is thus a good alternative. Baseline scores were generally similar across teams addressing different chronic illnesses and, like the original ACIC, the ACIC-S consistently showed improvement after intervention across CCM elements.

In line with earlier research on the ACIC, both the ACIC and the ACIC-S appear to be sensitive to intervention across different DMPs aimed at various chronic illnesses, helping teams focus their efforts on adopting evidence-based chronic care changes [17].

While Bonomi and colleagues [20] relied on group assessment of ACIC scores for a whole improvement team, we investigated individual assessment of each professional participating in the DMPs. The testing of theoretical associations between constructs can be analysed at the team level taking into account the hierarchical structure of the data for individuals nested within teams. As there is the potential for considerable variation within teams and since the main purpose of our study was to compare the psychometric properties of the ACIC in DMPs, we performed confirmatory factor analyses on the individual level. Ignoring the hierarchical structure of the data may lead to a worse fit of the model. The factor loadings found with the two methods (individual versus team level) will be similar in value [29,30].

For our sensitivity to change analyses we only had pre-post self-reported ACIC data for the four main components from the core teams of the 22 DMPs and thus could only test sensitivity to change of 'self-management support', 'delivery system design', 'decision support' and 'clinical information systems'. Since the ACIC is increasingly used to identify areas warranting improvement in chronic care and to evaluate whether care did indeed improve in such areas after intervention, the ACIC's sensitivity to change requires further substantiation. Unfortunately we were not able to conduct a 1 week retest of the instrument, further test-retest studies are necessary. Since it is time-consuming for professionals to implement the disease management programs and fill in the questionnaire during that time, we did not want to additionally burden them a week later with a second questionnaire. We also recommend testing the English version of the ACIC-S in other countries to ensure international validity. The responsiveness of the ACIC to improvement efforts notwithstanding, the presence of a control group (or control sites) would have strengthened our conclusions. While it is possible that completing the ACIC could act as an intervention based on the incidental education awarded by the survey itself, we do not think it likely given the difficulty in producing organizational change.

With these shortcomings in mind, we conclude that the psychometric properties of the ACIC and the ACIC-S are good and the ACIC-S is a promising alternate instrument to evaluate the level and nature of improvements made in DMPs.

## Ethical approval

The study was approved by the ethics committee of the Erasmus University Medical Centre of Rotterdam (September 2009).

## Competing interests

The authors declare that they have no competing interests.

## Authors' contributions

AN drafting the design for data gathering. JC, AN and AT were involved in acquisition of subjects and data. JC, AN and MS performed statistical analysis and interpretation of data. JC drafted the manuscript. AN, MS and AT helped drafting the manuscript and contributed to refinement. All authors contributed to the manuscript and have read and approved its final version.

## Appendix 1

1. ACIC Part 1; question 1) Overall organizational leadership in chronic illness care

2. ACIC Part 1; question 2) Organizational goals for chronic care

3. ACIC Part 1; question 3) Improvement strategy for chronic illness care

4. ACIC Part 1; question 4) Incentives and regulations for chronic illness care*

5. ACIC Part1; question 5) Senior leaders*

6. ACIC Part 1; question 6) Benefits*

7. ACIC Part 2; question 1) Linking patients to outside resources

8. ACIC Part 2; question 2) Partnership with community organizations

9. ACIC Part 2; question 3) Regional health plans

10. ACIC Part 3a; question 1) Assessment and documentation of self-management needs and activities

11. ACIC Part 3a; question 2) Self-management support

12. ACIC Part 3a; question 3) Addressing concerns of patients and families

13. ACIC Part 3a; question 4) Effective behavior change interventions and peer support*

14. ACIC Part 3b; question 1) Evidence-based guidelines

15. ACIC Part 3b; question 2) Involvement of specialists in improving primary care*

16. ACIC Part 3b; question 3) Providing education for chronic illness care

17. ACIC Part 3b; question 4) Informing patients about guidelines

18. ACIC Part 3c; question 1) Practice team functioning*

19. ACIC Part 3c; question 2) Practice team leadership*

20. ACIC Part 3c; question 3) Appointment system*

21. ACIC Part 3c; question 4) Follow-up

22. ACIC Part 3c; question 5) Planned visits for chronic illness care

23. ACIC Part 3c; question 6) Continuity of care

24. ACIC Part 3d; question 1) Registry (list of patients with specific conditions) *

25. ACIC Part 3d; question 2) Reminders to providers*

26. ACIC Part 3d; question 3) Feedback

27. ACIC Part 3d; question 4) Information about relevant subgroups of patients needing services

28. ACIC Part 3d; question 5) Patient treatment plans

29. ACIC Part 4; question 1) Informing patients about guidelines

30. ACIC Part 4; question 2) Information systems/registries*

31. ACIC Part 4; question 3) Community programs*

32. ACIC Part 4; question 4) Organizational planning for chronic illness care*

33. ACIC Part 4; question 5) Routine follow-up for appointments patient assessments and goal planning

34. ACIC Part 4; question 6) Guidelines for chronic illness care

* Items deleted after stepwise confirmatory factor analysis.

## Note

^1^Items were eliminated in the following order: 25, 24, 5, 6, 19, 20, 4, 31, 30, 13, 15, 32, and 18.
